# Vi Capsular Polysaccharide Produced by Recombinant *Salmonella enterica* Serovar Paratyphi A Confers Immunoprotection against Infection by *Salmonella enterica* Serovar Typhi

**DOI:** 10.3389/fcimb.2017.00135

**Published:** 2017-04-24

**Authors:** Kun Xiong, Chunyue Zhu, Zhijin Chen, Chunping Zheng, Yong Tan, Xiancai Rao, Yanguang Cong

**Affiliations:** ^1^Department of Microbiology, Third Military Medical UniversityChongqing, China; ^2^Outpatient Department of 95851 Unit of PLANanjing, China

**Keywords:** Vi capsular polysaccharide, enteric fever, *Salmonella enterica* serovar Typhi, *Salmonella enterica* serovar Paratyphi A, bivalent vaccine

## Abstract

Enteric fever is predominantly caused by *Salmonella enterica* serovar Typhi and *Salmonella enterica* serovar Paratyphi A, and accounts for an annual global incidence of 26.9 millions. In recent years, the rate of *S*. Paratyphi A infection has progressively increased. Currently licensed vaccines for typhoid fever, live Ty21a vaccine, Vi subunit vaccine, and Vi-conjugate vaccine, confer inadequate cross immunoprotection against enteric fever caused by *S*. Paratyphi A. Therefore, development of bivalent vaccines against enteric fever is urgently required. The immunogenic Vi capsular polysaccharide is characteristically produced in *S*. Typhi, but it is absent in *S*. Paratyphi A. We propose that engineering synthesis of Vi in *S*. Paratyphi A live-attenuated vaccine may expand its protection range to cover *S*. Typhi. In this study, we cloned the *viaB* locus, which contains 10 genes responsible for Vi biosynthesis, and integrated into the chromosome of *S*. Paratyphi A CMCC 50093. Two virulence loci, *htrA* and *phoPQ*, were subsequently deleted to achieve a Vi-producing attenuated vaccine candidate. Our data showed that, despite more than 200 passages, the *viaB* locus was stably maintained in the chromosome of *S*. Paratyphi A and produced the Vi polysaccharide. Nasal immunization of the vaccine candidate stimulated high levels of Vi-specific and *S*. Paratyphi A-specific antibodies in mice sera as well as total sIgA in intestinal contents, and showed significant protection against wild-type challenge of *S*. Paratyphi A or *S*. Typhi. Our study show that the Vi-producing attenuated *S*. Paratyphi A is a promising bivalent vaccine candidate for the prevention of enteric fever.

## Introduction

Enteric fever is a communicable and foodborne disease accounting for a global incidence of 26.9 million each year, and remains a serious public health issue in many developing countries (Buckle et al., 2012). Enteric fever is predominantly caused by the human restricted pathogens *Salmonella enterica* serovar Typhi and *S*. Paratyphi A (Crump and Mintz, 2010). *S*. Typhi is more common than *S*. Paratyphi A globally. However, in recent years, the morbidity of *S*. Paratyphi A-caused enteric fever has remarkably increased, particularly in some areas of Asia (Ochiai et al., 2005; Crump and Mintz, 2010; Sahastrabuddhe et al., 2013; Waddington et al., 2014). Currently licensed vaccines for enteric fever, oral live vaccine Ty21a, Vi subunit vaccine, and Vi-conjugate vaccine, are all based on *S*. Typhi, and confer inadequate cross immunoprotection against *S*. Paratyphi A infection (Sahastrabuddhe et al., 2013). Thus, vaccines against the infection of *S*. Paratyphi A are urgently required. Furthermore, because the epidemic areas of *S*. Typhi and *S*. Paratyphi A are largely overlapped (Crump and Mintz, 2010), a bivalent vaccine covering the two serovars is apparently a better choice than a monovalent vaccine in the control strategy of enteric fever.

Human infection with enteric fever pathogens normally occurs through the consumption of contaminated food or water (Dougan and Baker, 2014). Attenuated vaccines administered orally mimic the mucosal and systemic immune responses elicited by natural infection, and present multiple advantages. Particularly, the stimulation of mucosal immune response not only protects against disease but also reduces colonization and subsequent pathogen transmission to other susceptible hosts (Guzman et al., 2006). Taking advantages of live oral vaccine, we propose that engineering synthesis of a defined *S*. Typhi-specific protective antigen in *S*. Paratyphi A attenuated strain may generate a promising bivalent vaccine candidate.

Of the protective antigens in *S*. Typhi, the Vi capsular polysaccharide has been well-documented in term of biosynthesis and vaccine development (Hu et al., 2016). Vi is characteristically produced in most isolates of *S*. Typhi, whereas it is deficient in *S*. Paratyphi A (Hu et al., 2016). The biosynthesis of Vi is encoded by the *viaB* operon consisting of 10 genes that are associated with regulation of Vi expression (*tviA*), polysaccharide synthesis (*tviBCDE*), translocation, and cell attachment (*vexABCDE*) (Hu et al., 2016). The Vi polysaccharide plays a critical role in pathogenicity of *S*. Typhi by facilitating bacterial resistance to complement-mediated killing and phagocytosis (Robbins and Robbins, 1984; Liaquat et al., 2015; Hu et al., 2016). Vi also serves as a primary protective antigen of *S*. Typhi (Wong et al., 1974). Therefore, Vi subunit vaccine is now extensively used in preventing typhoid fever (Acharya et al., 1987; Klugman et al., 1987, 1996; Date et al., 2015). Given the defined role of the Vi polysaccharide in inducing protective immunity against *S*. Typhi infection, we attempted to construct a *S*. Paratyphi A attenuated strain with recombinant synthesis of the Vi polysaccharide.

In the present study, we introduced the *viaB* locus of *S*. Typhi into the chromosome of *S*. Paratyphi A CMCC50093. The Vi-producing strain was subsequently attenuated by deleting two virulence loci, *htrA* and *phoPQ*, to achieve a bivalent vaccine candidate. Our data show that *S*. Paratyphi A stably accommodated the *viaB* locus and produced the Vi polysaccharide. Nasal vaccination of the bivalent vaccine candidate resulted in striking serum and mucosal antibiody responses, and showed significant protective efficiency, in a mouse model, against challenges of the wild-type strain of *S*. Typhi, as well as the wild-type strain of *S*. Paratyphi A.

## Materials and methods

### Ethics statement

Animal experiments were carried out in accordance with the Guideline on the Humane Treatment of Laboratory Animals of the Ministry of Science and Technology of China. The protocol was reviewed and approved by the laboratory animal welfare and ethics committee of Third Military Medical University.

### Strains and plasmids

*S*. Typhi Ty2 and *S*. Paratyphi A CMCC50093 were purchased from The National Center for Medical Culture Collections (Beijing, China). *Escherichia coli* HB101 was a gift from Dr. Jing Wang of Third Military Medical University. *E*. *coli* S17-1/λpir was a gift from Dr. Victor de Lorenzo of the Centro Nacional de Biotecnologia CSIC, Spain. Plasmid pSTV28a (Takara, Dalian, China) was used for cloning the *viaB* operon. The suicide plasmid pYG4 constructed previously in our laboratory (Xiong et al., 2012), was used for integrating the *viaB* locus into the chromosome of *S*. Paratyphi A as well as subsequent deletions of virulence genes. Unless mentioned otherwise, bacteria were grown in an animal-product free medium containing 1% vegetable peptone, 0.5% yeast extract, and 1% NaCl. Antibiotics were used, as appropriate, in following concentrations: kanamycin 50 μg/ml; chloramphenicol 34 μg/ml. Primers used in the present study are listed in Table 1.

**Table 1:** **List of primers used in the present study**.

**Primers**	**Sequence**	**Note**
viaB-F	5′-cggggtaccagtatgacgttctgacggtt -3′	For amplification of the *viaB* locus
viaB-R	5′-cggggtaccattttcagctctgaagtaca -3′	
PNk1	5′-agatctggcgatgaaacgtgaaactc -3′	For amplification of up-stream sequence of *phoN*
PNk2	5′-gacataagtctggtacctgacgtctattgcgtggagtaaagaagaacgccc -3′	
PNk3	5′-cacgcaatagacgtcaggtaccagacttatgtctctgtaacatattccagg -3′	For amplification of down-stream sequence of *phoN*
PNk4	5′-catatgagcctatggctgggttt -3′	
PNk5	5′-agcagcacgcaatatttcaatgat -3′	For PCR-identification of the *viaB* locus
PNk6	5′-ggctcatatcgacatgatagatat -3′	
Pk1	5′- cccgggcccccttacaccacccagattga -3′	For amplification of up-stream sequence of *phoPQ*
Pk2	5′- cccgttataaatttggagtgtgaaggttattgcgtgctcttctcccttgtgttaac -3′	
Pk3	5′- ccccacgcaataaccttcacactccaaatttataacacatttctgcgcgttcttcc -3	For amplification of down-stream sequence of *phoPQ*
Pk4	5′- cccgagctcccggatcgctgtagtatgta -3′	
Pk5	5' -gggcatatggctattcgggtacgtcggcg-3'	For PCR-identification of *phoPQ*-deletion
Pk6	5' -gggcatatggtttagcggacgatgcgtaa-3'	
Hk1	5′- gggcatatgcttcagttagcgccttacag -3′	For amplification of up-stream sequence of *htrA*
Hk2	5′- gttataaatttggagtgtgaaggttattgcgtgtaatgtggtttttttcatgt -3′	
Hk3	5′- cacgcaataaccttcacactccaaatttataaccgtggtgatagttctattta -3′	For amplification of down-stream sequence of *htrA*
Hk4	5′- gggagatctgcgccacgcgaaaacgtagc -3′	
Hk5	5′- aaaaagtgatgccatcggtg -3′	For PCR-identification of *htrA*-deletion
Hk6	5′- aggctgattttgctgccgac -3′	

### Slide-agglutination assay

The synthesized Vi in tested bacteria was determined by slide-agglutination assay. Briefly, 10 μl of fresh cultures of the tested bacteria were dropped on a slide, and mixed thoroughly with an equal volume of Vi-diagnostic serum (Tianrun Bio-pharmaceutical Co., Ningbo, China). Aggregations formed within 1 min were recognized as positive reactions. Vi-encapsulated *S*. Typhi Ty2 strain was used as a positive control. *S*. Paratyphi A CMCC50093 (Vi^−^) was used as a negative control.

### Cloning of the *viaB* locus

The *viaB* locus (~13.9 kb) was PCR-amplified from genomic DNA of *S*. Typhi Ty2 by PrimeSTAR Max high fidelity DNA polymerase (Takara, Dalian, China) with primers viaB-F and viaB-R (Table 1). PCR was performed under the following conditions: 5 cycles of 98°C 10 s, 55°C 15 s, 72°C 10 min; 25 cycles of 98°C 10 s, 68°C 10 min. PCR product was gel-extracted using Wizard SV Gel and PCR clean-Up system (Promega, USA). After *Kpn* I-digestion, the amplified fragment was inserted into a low copy vector, pSTV28a, transformed into *E*. *coli* HB101 by electroporation and selected on agar plates containing 34 μg/ml chloramphenicol. Recombinant plasmids were identified with *Kpn* I-digestion, and then sent for DNA sequencing (BGI, China). Function of the *viaB* locus in *E*. *coli* was determined by slide-agglutination with Vi-diagnostic serum as described above. The identified recombinant plasmid was named pSTV28-viaB.

### Introducing the *viaB* locus into the chromosome of *S*. Paratyphi A

*S*. Paratyphi A CMCC50093 was utilized as the host strain in which the *phoN* gene was replaced with the cloned *viaB* locus. The *phoN* gene encodes an acidic phosphatase irrelevant to pathogenesis and is considered a neutral locus (Winter et al., 2010).

**Figure 1:**
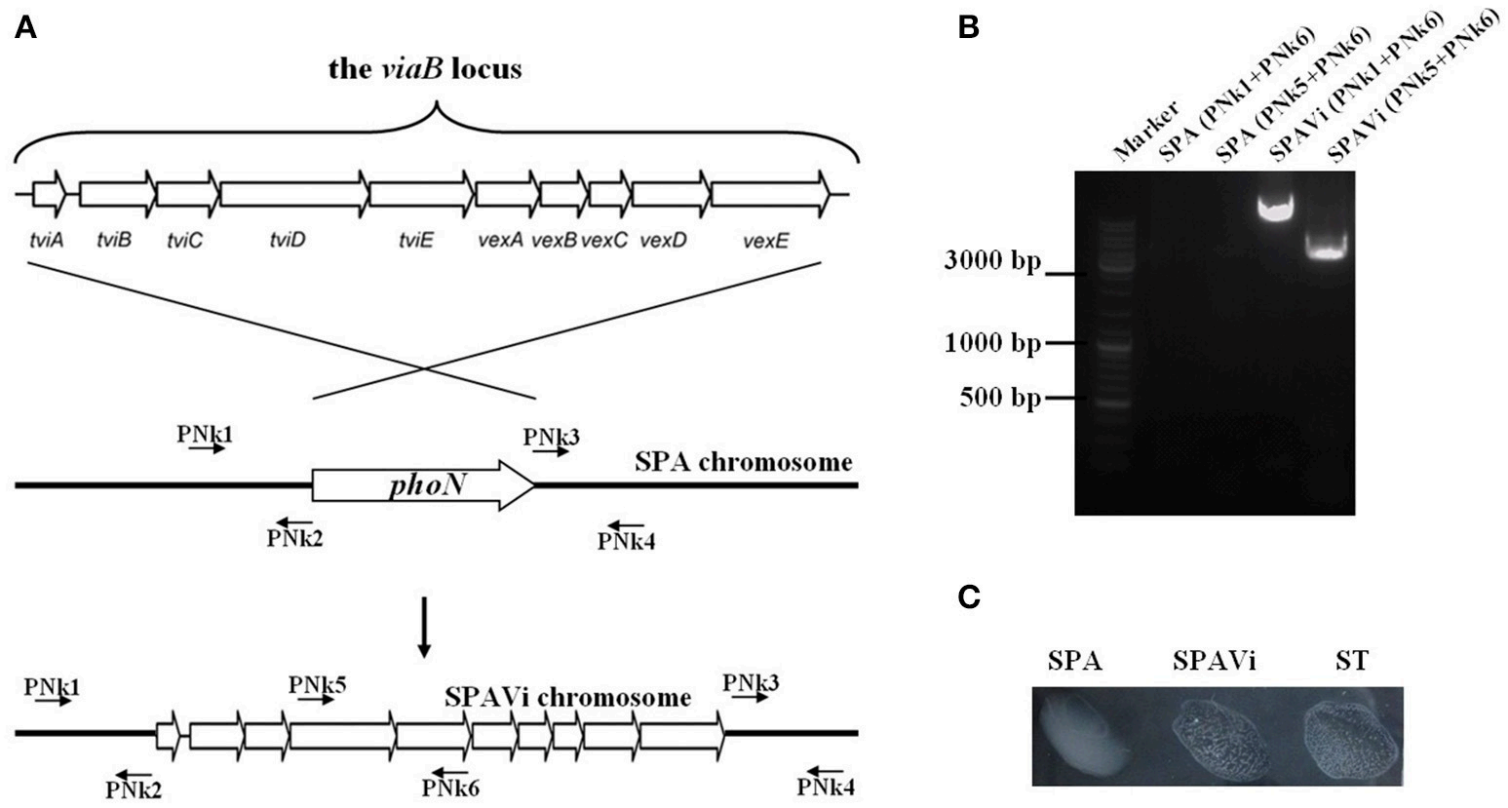
**Construction of the *viaB*-containing *Salmonella enterica* serovar Paratyphi A strain. (A)** Schematic diagram of strain construction. The *viaB* locus from *Salmonella enterica* serovar Typhi Ty2 was introduced into the chromosome of *S*. Paratyphi A by homologous replacement of *phoN*, a neutral gene of *Salmonella*. **(B)** PCR identification of the constructed strain. Relative positions of primers are indicated in schematic diagram. **(C)** The constructed strain, SPAVi, produces Vi leading to agglutination with Vi-specific antiserum. (SPA is abbreviation for *S*. Paratyphi A; SPAVi for *viaB*-containing *S*. Paratyphi A; ST for *S*. Typhi).

The suicide plasmid pYG4 was used as a vehicle for gene-replacement, which was manipulated as described previously (Xiong et al., 2012) and shown in Figure 1A. Briefly, upstream and downstream sequences of the *phoN* gene of *S*. Paratyphi A were amplified and combined by overlap PCR with primers PNk1 and PNk2, as well as PNk3 and PNk4. A *Kpn* I site was designed in 5′-terminals of PNk2 and PNk3, thus, a *Kpn* I site in the center of fusion fragment was generated by PCR. The PCR product was then digested with restriction enzymes of *Bgl* II and *Nde* I, and inserted into pYG4 to create pYG4-phoNUD. The *viaB*-containing fragment was cut from pSTV28-viaB by *Kpn* I, inserted into pYG4-phoNUD at the *Kpn* I site between the upstream and downstream sequences of *phoN*, and identified with *Kpn* I-digestion and slide-agglutination. The resultant plasmid was designated as pYG4-viaB.

The recombinant plasmid pYG4-viaB was transferred into *S*. Paratyphi A CMCC 50093 with electroporation, and selected for chromosomal plasmid-integrated strain with kanamycin resistance. The kanamycin-resistant strain was grown in liquid medium without antibiotics overnight and then counter-selected on agar plates supplemented with 5% of sucrose. The *S*. Paratyphi A strain with gene-replacement was screened by PCR, and further identified by slide-agglutination with the Vi antiserum. The resultant strain was named SPAVi.

### Deletion of *htrA* and *phoPQ* in SPAVi

To attenuate SPAVi, two virulence loci, *htrA* and *phoPQ*, were deleted. Gene-deletion was performed similarly to the manipulation of the gene-replacement except that a fusion DNA fragment consisting of the upstream and downstream sequences of the target gene was constructed in pYG4. The resultant Vi-producing attenuated strain was named SPA-VPH.

### Assessment of stability of the *viaB* locus in the chromosome of *S*. Paratyphi A

The *viaB*-bearing attenuated strain SPA-VPH was repeatedly passaged for over 200 times on agar plates. Vi-production of various generations was determined by dot immunoblotting. Fresh cultures of tested bacteria were inoculated into liquid medium at a ratio of 1:1,000 and incubated at 37°C for 4 h. The bacteria were harvested and resuspended in phosphate buffered saline (PBS, pH7.4) to an optical density (OD) of 0.8 at 600 nm. An equal volume of 10% SDS was added to lyse bacteria. Five microliters of 100-fold diluted lysates were added onto a nitrocellulose membrane. After drying at 60°C for 10 min, the membrane was blocked in PBST (PBS supplemented with 0.01% tween 20) containing 5% bovine serum albumin for 60 min, and then incubated in the Vi antiserum diluted at 1:3,000 in PBST for 30 min. After washing four times with PBST, secondary antibody (horseradish peroxidase conjugated goat anti-rabbit IgG, 1:5,000) was added and incubated for 30 min. The Vi polysaccharides were visualized with enhanced chemiluminescence detection kit (Thermo Scientific, USA).

### Assessment of osmotic regulation of the Vi synthesis in Vi-producing bacteria

Thirty microliters of bacterial suspension (0.05 OD_600_) of *S*. Typhi Ty2 or SPA-VPH were inoculated in 50 ml of Luria broth (LB) with varied NaCl concentrations from 0.1 to 0.7 M. After an overnight-incubation at 37°C with shaking at 200 rpm, the presence of Vi in the tested bacteria was detected by the slide-agglutination assay described above.

### Assessment of intracellular survival in THP-1 cell line

THP-1 cells were passaged in RPMI-1640 medium supplemented with 10% of fetal bovine serum and inoculated into 12-well plates at 5 × 10^5^ cells/well. Differentiation of THP-1 cells to macrophages was induced in the presence of phorbol myristate acetate (100 nM). Tested bacteria were then inoculated at 5 × 10^6^ colony forming units (CFU) each well. Following 2 h of incubation, cell monolayer was washed thrice with PBS. Extracellular bacteria were eliminated by incubating the cells in growth medium with 100 μg/ml gentamicin for 2 h. Infected cells were then incubated in the growth medium containing 50 μg/ml gentamicin in the remaining time. At 4, 8, and 14 h after bacterial inoculation, cell monolayer was washed with PBS, and lysed with 0.5% Trition X-100 to release intracellular bacteria. Lysates were serially diluted and inoculated on LB agar to enumerate bacteria.

### Determination of 50% lethal dose (LD_50_)

The LD_50_ was determined in a gastric mucin mouse model described elsewhere (Xiong et al., 2015). Briefly, fresh bacterial cultures were washed with PBS and diluted to 5 × 10^1^, 5 × 10^2^, 5 × 10^3^, 5 × 10^4^, 5 × 10^5^, 5 × 10^6^, and 5 × 10^7^ CFU/ml in 10% hog gastric mucin. BALB/c mice (10/group) were intraperitoneally injected with 0.5 ml of the bacterial suspensions, or with PBS as negative control. After inoculation, mice were observed for 72 h, and deaths were recorded.

### Bacterial burdens in the livers and spleens of infected mice

To further compare the virulence of *S*. Paratyphi A wild-type and SPA-VPH, bacterial loads in the livers and spleens of infected mice were assessed. Briefly, BALB/c mice aged 6–8 weeks (6/group) were infected by intraperitoneal injection with tested bacteria in absence of hog gastric mucin, at doses of 2.5 × 10^3^, 2.5 × 10^4^, and 2.5 × 10^5^ CFU per mouse. Infected mice were sacrificed on 7 day post-infection, and the livers and spleens were homogenized and serially diluted in sterile PBS. Bacterial numbers were determined by culture and colony counting.

### Immunoprotection against wild-type challenges of *S*. Typhi and *S*. Paratyphi A

Female BALB/c mice (10 per group) aged 6–8 weeks were nasally immunized with 2.0 × 10^9^ CFU of SPA-VPH, or PBS (negative control). On the 30 day after administration, immunized mice were intraperitoneally injected with 10-fold diluted bacterial suspensions of *S*. Typhi Ty2 or *S*. Paratyphi A wild-type strain from 2.5 × 10^1^ to 2.5 × 10^7^ CFU suspended in 10% gastric mucin. PBS-immunized mice were challenged with bacterial suspensions at doses from 2.5 × 10^1^ to 2.5 × 10^4^ CFU per mouse. After challenges, deaths of mice were monitored within 72 h.

### Determination of levels of anti-Vi IgG in the sera of immunized mice

BALB/c mice (6 per group) were nasally administered with SPA-VPH at 2.0 × 10^9^ CFU/mouse, or PBS. On the 0, 15, 30 day postvaccination, serum and intestinal samples were harvested. Levels of Vi-specific IgG in mice sera were determined with a Mouse S. Typhi Vi IgG ELISA kit (Alpha Diagnostic Intl. Inc., New Jersey, USA) according to the manufacturer's instructions.

### Determination of levels of *S*. Paratyphi A-specific IgGs in the sera of immunized mice

LPS and flagellin of *S*. Paratyphi A were isolated and purified as described elsewhere (Xiong et al., 2015). Levels of IgGs against LPS and flagella were measured by ELISA as described previously (Xiong et al., 2015).

### Determination of total sIgA in the intestinal contents of the immunized mice

Samples of intestinal contents of the immunized mice were collected at indicated time points. After 20-fold dilution, total sIgA levels were measured using a Mouse Secretory Immunoglobulin A ELISA Kit (CUSABIO, China) according to the manufacturer's instructions.

### Statistical analysis

LD_50_ was calculated by probit analysis using SPSS software. Student's unpaired *t*-test was used to compare group means. Survival rates were analyzed by Fisher's exact test. Values of *P* < 0.05 were regarded as statistically significant.

## Results

### Cloning of the *viaB* locus from *S*. Typhi to the chromosome of *S*. Paratyphi A

The entire *viaB* locus (~13.9 kb), which contains 10 genes accounting for the Vi synthesis and export, as well as the *tviA* promoter, was amplified by PCR with a high fidelity DNA polymerase, and inserted into pSTV28 creating pSTV28-viaB. Sequencing analysis of the resultant plasmid revealed no mutation in the *viaB* region. Consistently, the *viaB*-bearing plasmid can synthesize Vi in *E*. *coli* HB101, leading to positive agglutination with the Vi-specific antiserum. However, distinct expression stability of pSTV28-viaB was observed in various *E. coli* host cells. *E*. *coli* HB101 can stably host the Vi expression encoded by pSTV28-viaB, whereas *E*. *coli* DH5α can not. Several passages in chloramphenicol-containing liquid medium led to loss of the Vi synthesis in *E*. *coli* DH5α. Hence, *E*. *coli* HB101 cells were used as host bacteria for the replication of pSTV28-viaB in the present study.

From pSTV28-viaB, the *viaB* fragment was removed and inserted between the upstream and downstream sequences of *phoN* in the pYG4-phoNUD plasmid resulting in pYG4-viaB. By homologous recombination, the *phoN* gene in the chromosome of *S*. Paratyphi A CMCC50093 was replaced with the cloned *viaB* locus of pYG4-viaB (Figure 1A), resulting in a Vi-producing strain, SPAVi, determined by PCR and the slide-agglutination assay (Figures 1B,C).

### Construction of the *viaB*-containing attenuated strain of *S*. Paratyphi A

Two virulence loci, *htrA* and *phoPQ*, were deleted by homologous recombination to attenuate SPAVi (SFigure 1). The *htrA* gene encodes a stress response protease required for eliminating misfolded or damaged proteins in periplasmic space (Clausen et al., 2011; Skorko-Glonek et al., 2013). PhoPQ serves as a two-component regulatory system, which regulates the expression of several virulence genes, and plays a crucial role in pathogenesis (Miller et al., 1989). Both *htrA* and *phoPQ* have been extensively used in attenuating bacteria (Galán and curtiss, 1989; Chatfield et al., 1992; Karasova et al., 2009; Singh et al., 2013; Zhu et al., 2015).

**Figure 2:**
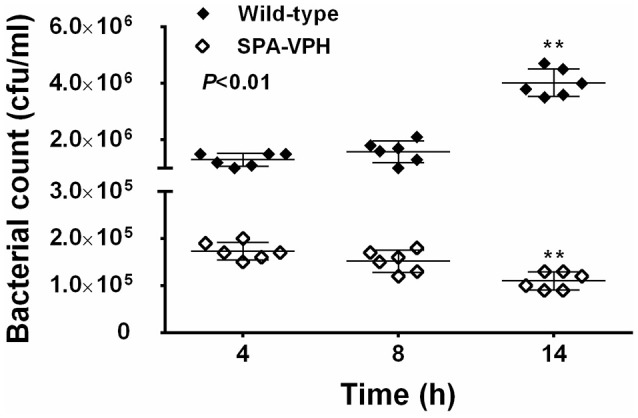
**Intracellular survival of *Salmonella enterica* serovar Paratyphi A wild-type and SPA-VPH in THP-1 cells**. Fresh cultures of tested bacteria, suspended in PBS, were inoculated to the differentiated THP-1 cells at a ratio of 10:1. After 2 h of incubation, extracellular bacteria were eliminated in the presence of 100 μg/ml gentamicin for 2 h. At the indicated time postinoculation, intracellular bacteria were released by addition of 0.5% Trition X-100 and quantitated by culture and colony counting. (^**^P < 0.01).

**Figure 3:**
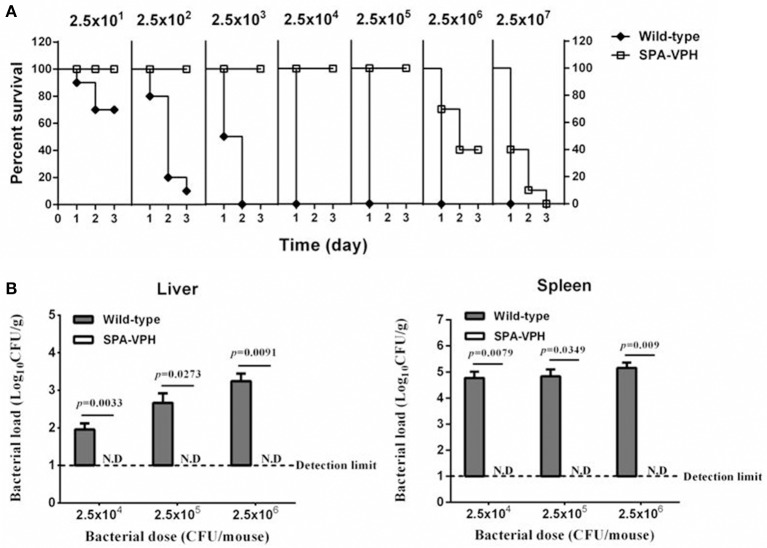
**Deletions of *phoPQ* and *htrA* in the *viaB*-containing *Salmonella enterica* serovar Paratyphi A led to dramatic attenuation in mice model**. **(A)** Survival curve of mice challenged with tested strains. The LD_50_ of *S*. Paratyphi A wild-type and SPA-VPH was assessed in a mucin mouse model. BALB/c mice were challenged with tested bacteria suspended in 10% hog gastric mucin by intraperitoneal injection at indicated doses. Deaths of mice were monitored within 72 h. **(B)** Bacterial burdens in organs of infected mice. Mice were intraperitoneally infected with *S*. Paratyphi A wild-type and SPA-VPH at indicated doses in the absence of hog gastric mucin. On the 7 day postinfection, bacterial persistence in the livers, and spleens of infected mice was assessed by culture and colony counting of tissue homogenates.

The resulting vaccine candidate, SPA-VPH, has reduced intracellular replication in THP-1 cells compared to the wild-type *S*. Paratyphi A (Figure 2). The LD_50_ of SPA-VPH assessed in the mucin mouse model is 1.91 × 10^6^ CFU, ~40,000 times higher than that of the wild-type strain (4.88 × 10^1^ CFU), showing a striking reduction in virulence (Figure 3A). In agreement with this, poor persistence of SPA-VPH in the livers and spleens of infected mice was observed (Figure 3B).

### The *viaB* locus is stably maintained in the chromosome of *S*. Paratyphi A

**Figure 4:**
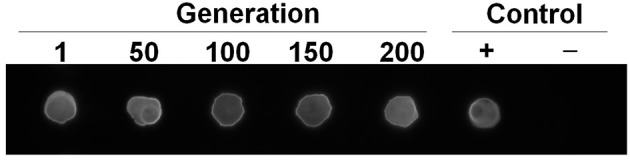
**Assessment of Vi-production in various generations of SPA-VPH**. Tested bacteria were suspended in PBS, and lysed by addition of equal volume of 10% SDS. Bacterial lysates were added onto a nitrocellulose membrane. The Vi polysaccharide binding to the membrane was detected with spot immunoblotting and visualized with enhanced chemiluminescence detection kit. Rabbit anti-Vi serum was used as the primary antibody. A goat anti-rabbit immunoglobulin G conjugated with horseradish peroxidase was used as the secondary antibody. Positive control is Vi-encapsulated *Salmonella enterica* serovar Typhi Ty2. Negative control is *Salmonella enterica* serovar Paratyphi A CMCC50093.

The *viaB* locus occurs in *S*. Typhi and *S*. Paratyphi C, two serovars genetically close to *S*. Paratyphi A, however, no natural Vi-encapsulated *S*. Paratyphi A has been isolated so far (Hu et al., 2016). This fact led us to speculate that an interfering factor may occur in *S*. Paratyphi A, which repels the stable existence of *viaB* in the chromosome of *S*. Paratyphi A. To address this concern, we examined the Vi synthesis in SPA-VPH, which underwent repeated passages. Despite more than 200 passages performed, no Vi-deficient phenotype was observed in these passaged strains (Figure 4). The data suggest that the *viaB* locus from *S*. Typhi can stably exist, and importantly, function under the genetic background of *S*. Paratyphi A.

The Vi capsular polysaccharide is under regulation in response to osmolarity in *S*. Typhi (Tartera and Metcalf, 1993; Pickard et al., 1994; Arricau et al., 1998). Vi-positive *S*. Typhi prefers to synthesize Vi in low to medium osmolarity environments with values lower than 0.3 M NaCl, whereas it turns down the Vi synthesis in high osmolarity environment (Pickard et al., 1994). To investigate whether the Vi expression of SPA-VPH is also regulated in a like manner, we assayed the Vi synthesis of SPA-VPH and *S*. Typhi Ty2 grown in LB broth with varied concentrations of NaCl. As shown in Table 2, cells of *S*. Typhi Ty2 and SPA-VPH agglutinated with Vi-specific antiserum when grown in media with NaCl concentrations of 0.1–0.4 M, whereas they no longer agglutinated at concentration of 0.7 M. At NaCl concentrations of 0.5 and 0.6 M, agglutinations were weak but observable; tiny aggregates were usually formed at 3–4 min after mixing of bacterial suspensions with the Vi antiserum. The data of our study slightly differs from those of a previous study by Pickard et al. (1994). In their investigation, *S*. Typhi bacteria cultured in the presence of 0.5–0.7 M NaCl did not agglutinate with the Vi antiserum. The minor difference likely reflects the distinct sensitivity of the antisera used in the two studies. However, it does not prevent our conclusion that SPA-VPH and *S*. Typhi share a same pattern of osmolarity regulation of the Vi synthesis.

**Table 2:** **Vi slide-agglutination reactions of *Salmonella enterica* serovar Typhi Ty2 and SPA-VPH grown in the presence of NaCl with varied concentrations**.

**NaCl concentration in LB (M)**	***S*. Typhi Ty2**	**SPA-VPH**
0.1	+	+
0.2	+	+
0.3	+	+
0.4	+	+
0.5	±	±
0.6	±	±
0.7	−	−

*nondetectable (−); positive (+); intermediate degree (±); LB (M), Luria Broth (mol/L)*.

### Nasal immunization of SPA-VPH induces high level of Vi-specific IgG in BALB/c mice

**Figure 5:**
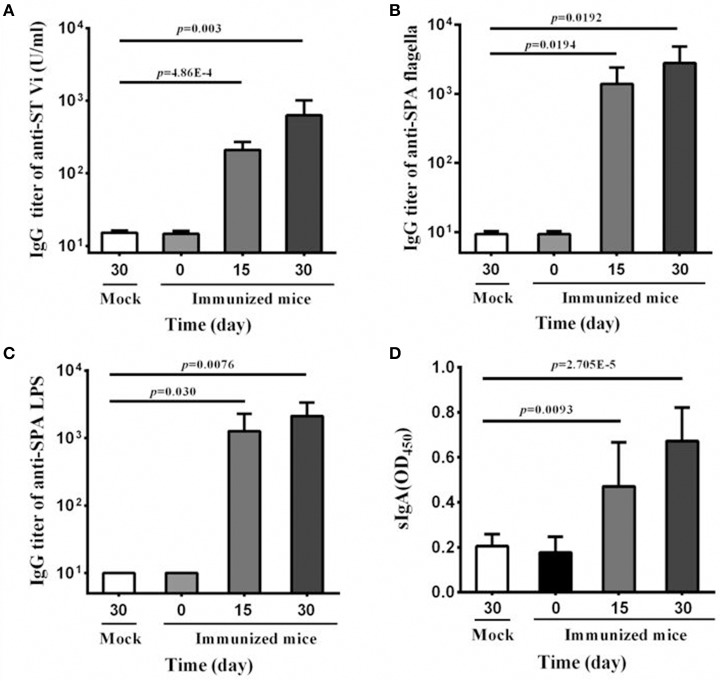
**Serum and mucosal antibody responses to intranasal immunization with SPA-VPH**. BALB/c mice were nasally administered with SPA-VPH at 2.0 × 10^9^ CFU/mouse. At indicated time points, samples of sera and intestinal contents were harvested. IgG and IgA levels were determined by ELISA. **(A)** Levels of IgG against the Vi of *Salmonella enterica* serovar Typhi in the mouse sera. **(B)** Levels of IgG against the flagellin of *Salmonella enterica* serovar Paratyphi A in the mouse sera. **(C)** Levels of IgG against the LPS of *S. Paratyphi* A in the mouse sera. **(D)** Levels of total sIgA against the cell lysate of *S. Paratyphi* A in the intestinal contents of the mice.

Due to its host restriction to humans, oral immunization elicits little or no immune response to serovar Typhi in mice model. In contrast, intranasal immunization is effective in inducing immune responses (Galen et al., 1997). Therefore, in the present study, the vaccine candidate was given in the intranasal route to examine the immunogenicity of SPA-VPH. As shown in Figure 5A, SPA-VPH immunization produced a significant rise in anti-Vi IgG level in mouse serum compared with mock-immunization, indicating that the Vi antigen produced by SPA-VPH is immunogenic in this animal model.

Since Vi-encapsulation may lead to poor exposure of other surface antigens, and consequently, low production of corresponding antibodies, we further detected the IgG levels of anti-LPS and anti-flagella in mice serum samples. Our results show that the immunization with SPA-VPH yielded high levels of IgGs against LPS and flagella, two important surface antigens of *S*. Paratyphi A (Figures 5B,C), suggesting that the Vi production does not abrogate the capability of eliciting immune responses by other surface antigens in SPA-VPH. Furthermore, we measured the total sIgA level in the intestinal contents of immunized mice. A significant increase of the total sIgA level was observed in the immunized mice compared with the mock-immunized mice (Figure 5D).

### Vaccination of SPA-VPH protects mice from the wild-type challenge by *S*. Typhi or *S*. Paratyphi A

**Figure 6:**
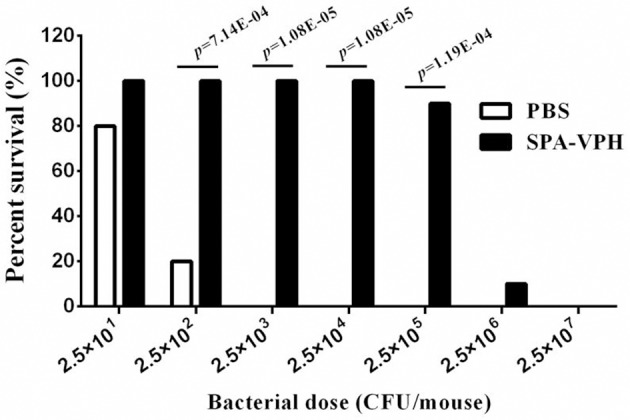
**Immunoprotection by nasal vaccination with SPA-VPH in BALB/c mice against challenge of *Salmonella enterica* serovar Paratyphi A wild-type**. BALB/c mice were nasally administered with SPA-VPH at 2.0 × 10^9^ CFU/mouse, or PBS as control. On the 30 day postvaccination, immunized mice were intraperitoneally challenged with 10-fold diluted bacterial suspensions of *S*. Paratyphi A wild-type from 2.5 × 10^1^to 2.5 × 10^7^ CFU suspended in 10% gastric mucin. PBS-immunized mice were challenged at doses from 2.5 × 10^1^ to 2.5 × 10^4^ CFU per mouse. After challenges, deaths of mice were monitored within 72 h. Survival percent of PBS-immunized mice at challenging doses from 2.5 × 10^5^ to 2.5 × 10^7^ are regarded as zero.

**Figure 7:**
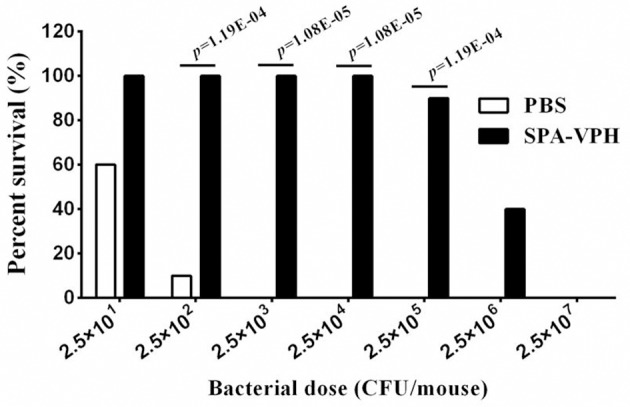
**Immunoprotection by nasal vaccination with SPA-VPH in BALB/c mice against challenge of *Salmonella enterica* serovar Typhi Ty2**. BALB/c mice were nasally administered with SPA-VPH at 2.0 × 10^9^ CFU/mouse, or PBS as control. On the 30 day postvaccination, immunized mice were intraperitoneally challenged with 10-fold diluted bacterial suspensions of *S*. Typhi Ty2 from 2.5 × 10^1^ to 2.5 × 10^7^ CFU suspended in 10% gastric mucin. PBS-immunized mice were challenged at doses from 2.5 × 10^1^ to 2.5 × 10^4^ CFU per mouse. After challenges, deaths of mice were monitored within 72 h. Survival percent of PBS-immunized mice at challenging doses from 2.5 × 10^5^ to 2.5 × 10^7^ are regarded as zero.

Given the satisfied immunogenicity of SPA-VPH described above, we subsequently assessed the immunoprotection efficacy of SPA-VPH nasally administered in mice. As shown in Figure 6, only 20% of PBS-vaccinated mice survived after a challenge of wild-type *S*. Paratyphi A at a dose of 2.5 × 10^2^ CFU/mouse. When challenged with wild-type *S*. Paratyphi A at doses exceeding 2.5 × 10^2^ CFU/mouse, all PBS-vaccinated mice died. In contrast, SPA-VPH-immunization yielded 90% of protection rate in tested mice with a challenging dose of 2.5 × 10^5^ CFU/mouse, and 100% of protection rate challenged at doses below 2.5 × 10^5^ CFU/mouse. Challenges of wild-type *S*. Typhi in immunized mice yielded results comparable to those of wild-type *S*. Paratyphi A (Figure 7). Only 10% of mice mock-immunized with PBS survived under a challenge of wild-type *S*. Typhi at 2.5 × 10^2^ CFU/mouse. At the challenge doses more than 2.5 × 10^2^ CFU/mouse, all tested mice died. In contrast, 90% of mice vaccinated with SPA-VPH survived after wild-type *S*. Typhi challenge at 2.5 × 10^5^ CFU/mouse, and no mice died below this dose.

Taken together, the animal experiments demonstrated that single nasal immunization with SPA-VPH in mice can yield adequate immunoprotection against infection caused by either *S*. Paratyphi A or *S*. Typhi.

## Discussion

Though *S*. Typhi and *S*. Paratyphi A belong to the same species and induce clinically indistinguishable syndromes, they are genetically and phenotypically distinct (McClelland et al., 2004; Näsström et al., 2014). The most apparent difference between *S*. Typhi and *S*. Paratyphi A is expression of the Vi capsular polysaccharide, which plays a critical role in the pathogenicity of *S*. Typhi, but is deficient in *S*. Paratyphi A (Hu et al., 2016). To the best of our knowledge, this is the first attempt at recombinant synthesis of the Vi polysaccharide in *S*. Paratyphi A. This study shows that *S*. Paratyphi A can stably accommodate the *viaB* operon, and synthesizes the Vi antigen *in vitro* and *in vivo*.

Enteric fever was a hyper-endemic infectious disease in China historically. However, the incidence rate of enteric fever has declined markedly in recent years, from 10–50 per 100,000 before 1990 to around 1 per 100,000 during the past 5 years, due to the rapid economic development in the past decades, the improvements in sanitation and water supply, as well as the large-scale use of the Vi polysaccharide vaccine. However, the incidence decrease shows regional differences. In 2012, 78% of enteric fever cases came from seven of the 33 provinces in China: Yunnan, Guizhou, Guangdong, Guangxi, Zhejiang, Hunan, and Xinjiang (Sun et al., 2013). In these regions with a total population over 373 million, enteric fever remains a major public health issue.

In 1990s, China began to produce and evaluate the Vi polysaccharide vaccine with the help of scientists from the National Institutes of Health in the United States (Jin, 2008). The locally produced Vi vaccine was proven to be effective with a protection rate of 70% in school-aged children and adults (Yang et al., 2001). Since then, the Vi vaccine has been using in large scale in China, and plays an important role in the control of enteric fever. For example, an increased coverage of the Vi vaccine in the hyper-endemic areas of Guangxi province led to a sharp decline in enteric fever incidence between 1998 and 1999 (Dong et al., 2010). However, during the same period, a serovar conversion from *S*. Typhi to *S*. Paratyphi A was also observed. No reported outbreaks in Guangxi were caused by *S*. Paratyphi A before 1998. After that, *S*. Paratyphi A accounted for most of enteric fever outbreaks (Dong et al., 2010). In several other provinces, the incidence of enteric fever caused by *S*. Paratyphi A also increased. In 2012, *S*. Paratyphi A accounted for 36.86% of total laboratory-diagnosed cases of enteric fever in China (Sun et al., 2013).

The increasing incidence of paratypoid fever in China as well as several other Asian countries generates a requirement for the development of vaccines against *S*. Paratyphi A infection (Sahastrabuddhe et al., 2013). The vaccines based on *S*. Typhi showed inadequate immunoprotection against the infection of *S*. Paratyphi A and vice versa (Simanjuntak et al., 1991; Wilde, 2007; Xiong et al., 2015). These facts indicate that level of the cross immunity between *S*. Typhi and *S*. Paratyphi A is low. Vi is well known for its roles in pathogenicity as well as inducing protective immunity against typhoid fever (Acharya et al., 1987; Klugman et al., 1987, 1996; Date et al., 2015; Hu et al., 2016). Therefore, the *viaB*-bearing *S*. Paratyphi A attenuated strain (SPA-VPH) constructed in the present study, which can stably synthesize the Vi antigen, were evaluated in its immunogenicity. Our data demonstrate that recombinantly produced Vi in *S*. Paratyphi A induced high level of Vi-specific IgG antibody in the sera of the immunized mice, moreover, it did not interfere the immunogenicity of other surface antigens, flagella and LPS, of *S*. Paratyphi A. As a result, nasal administration of SPA-VPH protected mice against the wild type challenge of either *S*. Typhi or *S*. Paratyphi A. These data show that *S*. Paratyphi A strains with Vi-producing capability have potential for being developed to bivalent vaccines.

For an attenuated live vaccine, the optimal balance between safety and immunogenicity must be achieved. Vi is a well-known virulence factor and plays a critical role in pathogenicity of *S*. Typhi (Hu et al., 2016). Therefore, the safety of the attenuated Vi-positive *S*. Paratyphi A strain must be evaluated more carefully in clinical trials. Moreover, previous data showed that native or constitutive expressed Vi on live oral typhoid vaccines elicited poor immune responses in human body (Tacket et al., 1991, 2004). Thus, further modifications of the current vaccine candidate are needed before clinical trials. For instance, replacement of the native promoter of *tviA*, which governs the transcription of the *viaB* operon, with an *in vivo* inducible promoter may improve the immunogenicity of Vi, as shown in work by Janis et al. (2011).

In summary, our data demonstrate that *S*. Paratyphi A can accommodate the *viaB* locus from *S*. Typhi, and stably expresses the Vi antigen. The osmoregulation of Vi in *S*. Paratyphi A is analogous to that in *S*. Typhi. The Vi-capsulated *S*. Paratyphi A strain confers an adequate immunoprotection against the infection of *S*. Typhi, which may contribute to the development of bivalent enteric fever vaccine.

## Author contributions

YC and XR designed the experiments. KX, CZ, YC, ZC, CZ, and YT performed the experiments. YC wrote the manuscript. All authors reviewed the results and approved the final manuscript.

### Conflict of interest statement

The authors declare that the research was conducted in the absence of any commercial or financial relationships that could be construed as a potential conflict of interest.

## References

[B1] Acharya I. L. Lowe C. U. Thapa R. Gurubacharya V. L. Shrestha M. B. Cadoz M.. (1987). Prevention of typhoid fever in Nepal with the Vi capsular polysaccharide of *Salmonella typhi*. A preliminary report. N. Engl. J. Med. 317, 1101–1104. 10.1056/NEJM198710293171801 3657877

[B2] Arricau N. Hermant D. Waxin H. Ecobichon C. Duffey P. S. Popoff M. Y. (1998). The RcsB-RcsC regulatory system of *Salmonella typhi* differentially modulates the expression of invasion proteins, flagellin and Vi antigen in response to osmolarity. Mol. Microbiol. 29, 835–850. 10.1046/j.1365-2958.1998.00976.x 9723922

[B3] Buckle G. C. Walker C. L. Black R. E. (2012). Typhoid fever and paratyphoid fever: systematic review to estimate global morbidity and mortality for 2010. J. Glob. Health 2:010401. 10.7189/jogh.01.010401 23198130 PMC3484760

[B4] Chatfield S. N. Strahan K. Pickard D. Charles I. G. Hormaeche C. E. Dougan G. (1992). Evaluation of *Salmonella typhimurium* strains harbouring defined mutations in *htrA* and *aroA* in the murine salmonellosis model. Microb. Pathog. 12, 145–151. 10.1016/0882-4010(92)90117-7 1584006

[B5] Clausen T. Kaiser M. Huber R. Ehrmann M. (2011). HTRA proteases: regulated proteolysis in protein quality control. Nat. Rev. Mol. Cell Biol. 12, 152–162. 10.1038/nrm3065 21326199

[B6] Crump J. A. Mintz E. D. (2010). Global trends in typhoid and paratyphoid Fever. Clin. Infect. Dis. 50, 241–246. 10.1086/649541 20014951 PMC2798017

[B7] Date K. A. Bentsi-Enchill A. Marks F. Fox K. (2015). Typhoid fever vaccination strategies. Vaccine 33(Suppl. 3), C55–C61. 10.1016/j.vaccine.2015.04.028 25902360 PMC10644681

[B8] Dong B. Q. Yang J. Wang X. Y. Gong J. von Seidlein L. Wang M. L.. (2010). Trends and disease burden of enteric fever in Guangxi province, China, 1994–2004. Bull. World Health Organ. 88, 689–696. 10.2471/BLT.09.069310 20865074 PMC2930361

[B9] Dougan G. Baker S. (2014). *Salmonella enterica* serovar Typhi and the pathogenesis of typhoid fever. Annu. Rev. Microbiol. 68, 317–336. 10.1146/annurev-micro-091313-103739 25208300

[B10] Galán J. E. curtiss R. III. (1989). Virulence and vaccine potential of phoP mutants of *Salmonella typhimurium*. Microb. Pathog. 6, 433–443. 10.1016/0882-4010(89)90085-5 2671582

[B11] Galen J. E. Gomez-Duarte O. G. Losonsky G. A. Halpern J. L. Lauderbaugh C. S. Kaintuck S.. (1997). A murine model of intranasal immunization to assess the immunogenicity of attenuated *Salmonella typhi* live vector vaccines in stimulating serum antibody responses to expressed foreign antigens. Vaccine 15, 700–708. 10.1016/S0264-410X(96)00227-7 9178472

[B12] Guzman C. A. Borsutzky S. Griot-Wenk M. Metcalfe I. C. Pearman J. Collioud A.. (2006). Vaccines against typhoid fever. Vaccine 24, 3804–3811. 10.1016/j.vaccine.2005.07.111 16278037

[B13] Hu X. Chen Z. Xiong K. Wang J. Rao X. Cong Y. (2016). Vi capsular polysaccharide: synthesis, virulence, and application. Crit. Rev. Microbiol. 10.1080/1040841x.2016.1249335. [Epub ahead of print]. 27869515

[B14] Janis C. Grant A. J. McKinley T. J. Morgan F. J. John V. F. Houghton J.. (2011). *In vivo* regulation of the Vi antigen in *Salmonella* and induction of immune responses with an *in vivo*-inducible promoter. Infect. Immun. 79, 2481–2488. 10.1128/IAI.01265-10 21402763 PMC3125847

[B15] Jin Y. (2008). Enteric fever in south China: Guangxi province. J. Infect. Dev. Ctries 2, 283–288. 10.3855/jidc.223 19741290

[B16] Karasova D. Sebkova A. Vrbas V. Havlickova H. Sisak F. Rychlik I. (2009). Comparative analysis of *Salmonella enterica* serovar Enteritidis mutants with a vaccine potential. Vaccine 27, 5265–5270. 10.1016/j.vaccine.2009.06.060 19577637

[B17] Klugman K. P. Gilbertson I. T. Koornhof H. J. Robbins J. B. Schneerson R. Schulz D.. (1987). Protective activity of Vi capsular polysaccharide vaccine against typhoid fever. Lancet 2, 1165–1169. 10.1016/S0140-6736(87)91316-X 2890805

[B18] Klugman K. P. Koornhof H. J. Robbins J. B. Le Cam N. N. (1996). Immunogenicity, efficacy and serological correlate of protection of *Salmonella typhi* Vi capsular polysaccharide vaccine three years after immunization. Vaccine 14, 435–438. 10.1016/0264-410X(95)00186-5 8735556

[B19] Liaquat S. Sarwar Y. Ali A. Haque A. (2015). Comparative growth analysis of capsulated (Vi+) and acapsulated (Vi-) *Salmonella typhi* isolates in human blood. EXCLI J. 14, 213–219. 10.17179/excli2014-674 26417360 PMC4553862

[B20] McClelland M. Sanderson K. E. Clifton S. W. Latreille P. Porwollik S. Sabo A.. (2004). Comparison of genome degradation in Paratyphi, A., and Typhi, human-restricted serovars of *Salmonella enterica* that cause typhoid. Nat. Genet. 36, 1268–1274. 10.1038/ng1470 15531882

[B21] Miller S. I. Kukral A. M. Mekalanos J. J. (1989). A two-component regulatory system (*phoP phoQ*) controls *Salmonella typhimurium* virulence. Proc. Natl. Acad. Sci. U.S.A. 86, 5054–5058. 10.1073/pnas.86.13.5054 2544889 PMC297555

[B22] Näsström E. Vu Thieu N. T. Dongol S. Karkey A. Voong Vinh P. Ha Thanh T.. (2014). Salmonella Typhi and Salmonella Paratyphi A elaborate distinct systemic metabolite signatures during enteric fever. Elife 3:e03100. 10.7554/eLife.03100 24902583 PMC4077204

[B23] Ochiai R. L. Wang X. von Seidlein L. Yang J. Bhutta Z. A. Bhattacharya S. K.. (2005). *Salmonella paratyphi* A rates, Asia. Emerg. Infect. Dis. 11, 1764–1766. 10.3201/eid1111.050168 16318734 PMC3367370

[B24] Pickard D. Li J. Roberts M. Maskell D. Hone D. Levine M. (1994). Characterization of defined *ompR* mutants of *Salmonella typhi*: ompR is involved in the regulation of Vi polysaccharide expression. *Infect*. Immun. 62, 3984–3993. 10.1128/iai.62.9.3984-3993.1994 PMC303057 8063417

[B25] Robbins J. D. Robbins J. B. (1984). Reexamination of the protective role of the capsular polysaccharide (Vi antigen) of *Salmonella typhi*. J. Infect. Dis. 150, 436–449. 10.1093/infdis/150.3.436 6207249

[B26] Sahastrabuddhe S. Carbis R. Wierzba T. F. Ochiai R. L. (2013). Increasing rates of *Salmonella* Paratyphi, A., and the current status of its vaccine development. Expert Rev. Vaccines 12, 1021–1031. 10.1586/14760584.2013.825450 24053396

[B27] Simanjuntak C. H. Paleologo F. P. Punjabi N. H. Darmowigoto R. Soeprawoto Totosudirjo H.. (1991). Oral immunisation against typhoid fever in Indonesia with Ty21a vaccine. Lancet 338, 1055–1059. 10.1016/0140-6736(91)91910-M 1681365

[B28] Singh B. R. Chandra M. Hansda D. Alam J. Babu N. Siddiqui M. Z.. (2013). Evaluation of vaccine candidate potential of deltaaroA, deltahtrA and deltaaroAdeltahtrA mutants of *Salmonella enterica* subspecies enterica serovar Abortusequi in guinea pigs. Indian J. Exp. Biol. 51, 280–287. 24195347

[B29] Skorko-Glonek J. Zurawa-Janicka D. Koper T. Jarzab M. Figaj D. Glaza P.. (2013). HtrA protease family as therapeutic targets. Curr. Pharm. Des. 19, 977–1009. 10.2174/1381612811319060003 23016688

[B30] Sun J. L. Zhang J. Ma H. L. Chang Z. R. (2013). [Epidemiological features of typhoid/paratyphoid fever in provinces with high incidence rate and in the whole country, in 2012]. Zhonghua Liu Xing Bing Xue Za Zhi 34, 1183–1188. 10.3760/cma.j.issn.0254-6450.2013.012.007 24518016

[B31] Tacket C. O. Losonsky G. Taylor D. N. Baron L. S. Kopecko D. Cryz S.. (1991). Lack of immune response to the Vi component of a Vi-positive variant of the *Salmonella typhi* live oral vaccine strain Ty21a in human studies. J. Infect. Dis. 163, 901–904. 10.1093/infdis/163.4.901 2010645

[B32] Tacket C. O. Pasetti M. F. Sztein M. B. Livio S. Levine M. M. (2004). Immune responses to an oral typhoid vaccine strain that is modified to constitutively express Vi capsular polysaccharide. J. Infect. Dis. 190, 565–570. 10.1086/421469 15243933

[B33] Tartera C. Metcalf E. S. (1993). Osmolarity and growth phase overlap in regulation of *Salmonella typhi* adherence to and invasion of human intestinal cells. Infect. Immun. 61, 3084–3089. 8514418 10.1128/iai.61.7.3084-3089.1993 PMC280966

[B34] Waddington C. S. Darton T. C. Pollard A. J. (2014). The challenge of enteric fever. J. Infect. 68(Suppl. 1), S38–S50. 10.1016/j.jinf.2013.09.013 24119827

[B35] Wilde H. (2007). Enteric fever due to *Salmonella typhi* and *paratyphi A* a neglected and emerging problem. Vaccine 25, 5246–5247. 10.1016/j.vaccine.2007.04.053 17560692

[B36] Winter S. E. Winter M. G. Godinez I. Yang H. J. Russmann H. Andrews-Polymenis H. L.. (2010). A rapid change in virulence gene expression during the transition from the intestinal lumen into tissue promotes systemic dissemination of *Salmonella*. PLoS Pathog. 6:e1001060. 10.1371/journal.ppat.1001060 20808848 PMC2924370

[B37] Wong K. H. Feeley J. C. Northrup R. S. Forlines M. E. (1974). Vi antigen from *Salmonella typhosa* and immunity against typhoid fever. I. Isolation and immunologic properties in animals. Infect. Immun. 9, 348–353. 4205947 10.1128/iai.9.2.348-353.1974 PMC414808

[B38] Xiong K. Chen Z. Xiang G. Wang J. Rao X. Hu F.. (2012). Deletion of *yncD* gene in *Salmonella enterica* ssp. enterica serovar Typhi leads to attenuation in mouse model. FEMS Microbiol. Lett. 328, 70–77. 10.1111/j.1574-6968.2011.02481.x 22150228

[B39] Xiong K. Chen Z. Zhu C. Li J. Hu X. Rao X.. (2015). Safety and immunogenicity of an attenuated *Salmonella enterica* serovar Paratyphi A vaccine candidate. Int. J. Med. Microbiol. 305, 563–571. 10.1016/j.ijmm.2015.07.004 26239100

[B40] Yang H. H. Wu C. G. Xie G. Z. Gu Q. W. Wang B. R. Wang L. Y.. (2001). Efficacy trial of Vi polysaccharide vaccine against typhoid fever in south-western China. Bull. World Health Organ. 79, 625–631. 11477965 PMC2566475

[B41] Zhu C. Xiong K. Chen Z. Hu X. Li J. Wang Y.. (2015). Construction of an attenuated *Salmonella enterica* serovar Paratyphi A vaccine strain harboring defined mutations in htrA and yncD. Microbiol. Immunol. 59, 443–451. 10.1111/1348-0421.12276 26084199

