# Supplementary material for: Vi Capsular Polysaccharide Produced by Recombinant Salmonella enterica Serovar Paratyphi A Confers Immunoprotection against Infection by Salmonella enterica Serovar Typhi
Source: Front Cell Infect Microbiol. 2017 Apr 24;7:135. doi: 10.3389/fcimb.2017.00135 (PMC5401900; doi:10.3389/fcimb.2017.00135)
Supplement: Supplementary file 1 [file Image1.PDF]

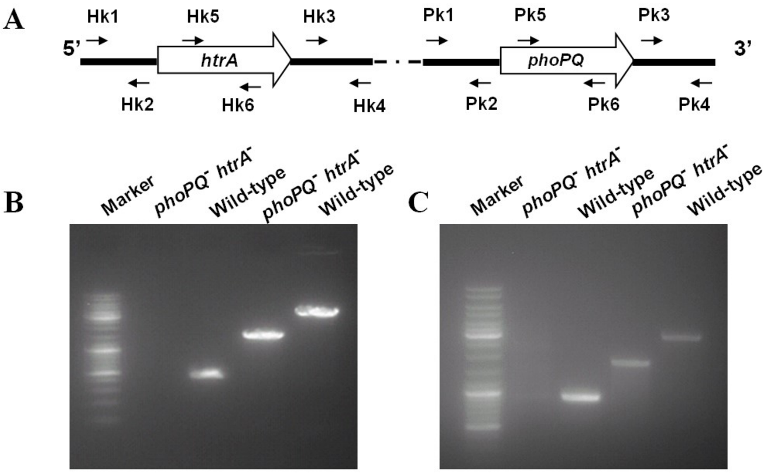


SFigure 1. Identification of deletions of *phoPQ* and *htrA* by PCR. (A) Relative positions of primers used in strain construction and identification. (B) PCR identification of *htrA*-deletion. Lane 1: Molecular weight marker; Lane 2&3: PCR products with primers Hk5 and Hk6; Lane 4&5: PCR products with primers Hk1 and Hk4. (C) PCR identification of *phoPQ*-deletion. Lane 1: Molecular weight marker; Lane 2&3: PCR products with primers Pk5 and Pk6; Lane 4&5: PCR products with primers Pk1 and Pk4.
